# Computed tomography chest imaging offers no advantage over chest X-ray in the initial assessment of gestational trophoblastic neoplasia

**DOI:** 10.1038/s41416-020-01206-8

**Published:** 2020-12-16

**Authors:** Victoria L. Parker, Matthew C. Winter, Elspeth Whitby, William A. E. Parker, Julia E. Palmer, John A. Tidy, Allan A. Pacey, Barry W. Hancock, Robert F. Harrison

**Affiliations:** 1 Department of Oncology and Metabolism, The Medical School, The University of Sheffield, Beech Hill Road, Sheffield, S10 2RX UK; 2 Sheffield Centre for Trophoblastic Disease, Weston Park Cancer Centre, Sheffield Teaching Hospitals NHS Foundation Trust, Whitham Road, Sheffield, S10 2SJ UK; 3 School of Medicine, The University of Sheffield, Beech Hill Road, Sheffield, S10 2RX UK; 4 Department of Gynaecological Oncology, Royal Hallamshire Hospital, Sheffield Teaching Hospitals NHS Foundation Trust, Room H18, Glossop Road, Sheffield, S10 2JF UK; 5 Department of Automatic Control and Systems Engineering, The University of Sheffield, Mappin Street, Sheffield, S1 3JD UK

**Keywords:** Outcomes research, Endometrial cancer

## Abstract

**Background:**

The International Federation of Gynaecology and Obstetrics (FIGO) score identifies gestational trophoblastic neoplasia (GTN) patients as low- or high-risk of single-agent chemotherapy resistance (SACR). Computed tomography (CT) has greater sensitivity than chest X-ray (CXR) in detecting pulmonary metastases, but effects upon outcomes remain unclear.

**Methods:**

Five hundred and eighty-nine patients underwent both CXR and CT during GTN assessment. Treatment decisions were CXR based. The number of metastases, risk scores, and risk category using CXR versus CT were compared. CT-derived chest assessment was evaluated as impact upon treatment decision compared to patient outcome, incidence of SACR, time-to-normal human chorionic gonadotrophin hormone (TNhCG), and primary chemotherapy resistance (PCR).

**Results:**

Metastasis detection (*p* < 0.0001) and FIGO score (*p* = 0.001) were higher using CT versus CXR. CT would have increased FIGO score in 188 (31.9%), with 43 re-classified from low- to high-risk, of whom 23 (53.5%) received curative single-agent chemotherapy. SACR was higher when score (*p* = 0.044) or risk group (*p* < 0.0001) changed. Metastases on CXR (*p* = 0.019) but not CT (*p* = 0.088) lengthened TNhCG. Logistic regression analysis found no difference between CXR (area under the curve (AUC) = 0.63) versus CT (AUC = 0.64) in predicting PCR.

**Conclusions:**

CT chest would improve the prediction of SACR, but does not influence overall treatment outcome, TNhCG, or prediction of PCR. Lower radiation doses and cost mean ongoing CXR-based assessment is recommended.

## Background

Gestational trophoblastic neoplasia (GTN) is generally classified using the International Federation of Gynaecology and Obstetrics (FIGO) scoring system, identifying patients at low-risk (score ≤ 6) or high-risk (score ≥ 7) of resistance to single-agent chemotherapy.^1^ The system can be applied to patients diagnosed with GTN after a complete or partial hydatidiform mole, invasive mole, or choriocarcinoma, but cannot be used for the rarer tumour subtypes of placental-site- (PSTT) or epithelioid-trophoblastic tumour (ETT) due to their differing behaviour and characteristics.^2–6^ In the United Kingdom (UK), women with low-risk GTN receive single-agent methotrexate, while high-risk patients receive multi-agent chemotherapy, usually EMA-CO (Etoposide, Methotrexate, Actinomycin D/Cyclophosphamide and Vincristine).^5^

The FIGO scoring system uses chest X-ray (CXR) as standard to assess pulmonary metastases. In UK practice, pulmonary metastases are evaluated on CXR, with computed tomography (CT) only performed if there is an uncertainty over the presence of lesions on CXR.^5^ As previously acknowledged by the FIGO committee,^7,8^ CT chest offers advantages over CXR in terms of increased detection of pulmonary metastases, yet the impact upon treatment decisions and outcome is unknown, leading to long-standing controversy regarding its routine use in the assessment of GTN.^7,9–14^ One issue concerns whether pulmonary metastases detectable only on CT are of clinical importance, with some studies concluding that they are a significant prognostic factor for single-agent chemotherapy resistance and longer time to achieve first normal human chorionic gonadotrophin hormone (TNhCG),^7,11,15^ while others disagree.^10,13,14,16^ This controversy is hampered by the study of differing patient groups (low-risk-only, low- and high-risk patients), with varying outcome measures, such as chemotherapy resistance, time to remission, or disease recurrence. In several previous studies, a separate analysis of patients with metastases detectable only on CT has not been performed, making conclusions difficult to deduce.^7,9,13,15,16^ Given the rare nature of GTN, and the use of incomplete, retrospective datasets, accurate statistical comparisons are problematic.^7,9,16^

To resolve the controversy regarding the role of CT chest in the assessment of GTN, we examined a large UK dataset of patients from a leading Gestational Trophoblastic Disease Centre. CT-derived chest assessment was evaluated in four different ways: (i) the effect upon treatment decisions compared to actual patient outcome; (ii) observed incidence of single-agent chemotherapy resistance; (iii) the effect upon TNhCG; a surrogate marker for remission;^7^ and (iv) the prediction of primary chemotherapy resistance in all treated patients. Separate secondary analyses were performed: (1) upon groups (i)–(iii) to study patients with chest metastases detectable only on CT; and (2) to analyse the incidence of relapse and death in the dataset. Treatment decisions were based upon CXR-derived assessment of GTN, and treatment changes indicated by CT were not carried out.

## Methods

### Data collection

All patients diagnosed with GTN and referred to the Sheffield Trophoblastic Centre between January 1973 and April 2019 (*n* = 1294) were included in this study. Patients were excluded if they had: (i) histology inconsistent with Gestational Trophoblastic Disease following review by specialist pathologists at the Sheffield Trophoblastic Centre; (ii) were not treated (with either chemotherapy or surgery beyond the initial uterine evacuations); (iii) diagnosed with rare histological subtypes of PSTT or ETT; and (iv) duplicate data entries. Included patients had: (i) undergone both a CXR and CT chest during initial investigations for GTN; (ii) a complete FIGO score, including a breakdown of the eight contributing components; and (iii) outcome data regarding single-agent and primary chemotherapy response (treatment resistance (TR) versus complete response (CR)). Single-agent chemotherapy involved patients categorised as low-risk, whereas primary chemotherapy was defined as first-line treatment in low- or high-risk patients, and as such could be single or multi-agent. TR to single-agent or primary chemotherapy was defined as a rise in ≥2 serial serum hCG levels over 4 weeks, or ≥3 consecutive hCG readings that did not fall as expected (by ~25%) over the same time period.^17^ Relapse was defined as ≥2 rising serial serum hCG levels in the absence of a new pregnancy or alternative explanation, following ≥6 weeks of normal serum hCG levels following the completion of chemotherapy to initially achieve CR.^18^ Treatment decisions were entirely based on CXR-derived assessment of GTN. Selection and details of chemotherapy regimens can be found in Supplementary Table S1.

CXR and CT chest images were reviewed and re-reported when the original report did not comment upon the exact number and size of metastases. In line with the criteria previously reported by Price et al.,^9^ radiographic features deemed to represent metastases included solid, well-defined lesions of a round shape in the proximity of, or at the end of, a vessel, with evidence of surrounding haemorrhage (ground-glass opacification). Multiple small lesions were assumed to be metastases, while lesions suggestive of a granuloma (calcified, spiculated, and in relation to an airway) or benign lesion (oval in shape, thickened interlobular septa) were excluded. Lesions that remained uncertain in nature were reviewed upon serial imaging, and those that did not resolve with treatment were deemed to be non-metastatic and excluded from the analysis. Lesions of all sizes that satisfied the above criteria were included and counted, to the smallest detectable size of 1 mm.

### Statistical analysis

Raw data (total number of metastases, FIGO score, and TNhCG) were checked for normality (Shapiro–Wilk test) prior to statistical analysis. Wilcoxon matched-pairs signed-rank test was used to compare the total number of metastases detected on CXR versus CT. Paired nominal data in terms of FIGO risk category (low-risk versus high-risk) and response to single-agent chemotherapy (TR versus CR) were compared using McNemar’s test. Fisher’s exact test was used to compare rates of single-agent chemotherapy resistance among patients whose total FIGO score and risk category had changed as a result of CT-derived chest imaging. Differences in TNhCG were investigated using the log-rank Mantel–Cox test. Finally, binomial logistic regression analyses were used for the prediction of TR to primary chemotherapy using multiple categorical or continuous variables, with no assumption of independence between these variables. Statistical analyses were performed in GraphPad Prism (version 8, San Diego, CA, USA) and MatLab (version R2018b, Natick, MA, USA).

### Results

**Fig. 1:**
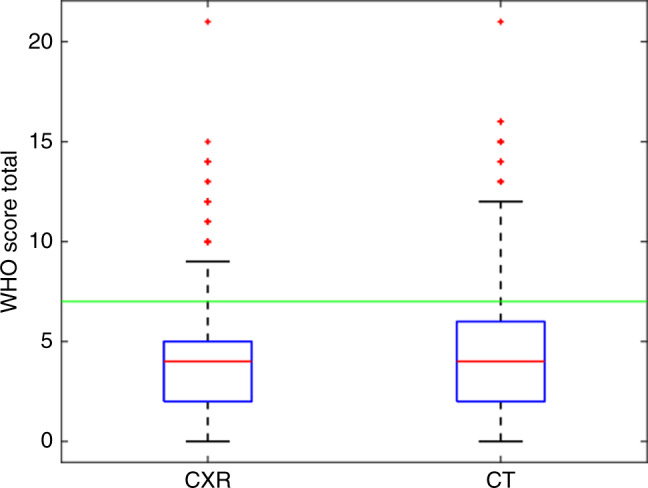
Box and whisker plot comparing the FIGO scores calculated using CXR- versus CT-based imaging of pulmonary metastases. The threshold line delineates a FIGO score of 7; the cut-off for categorising patients as low- versus high-risk. *FIGO* International Federation of Gynaecology and Obstetrics, *CXR* chest X-ray, *CT* computerised tomography (chest).

Of the 1294 patients included, 589 met the inclusion criteria (CONSORT diagram and Supplementary Table S2). The total number of metastases detected on CT chest was significantly higher than on CXR (Wilcoxon matched-pairs signed-rank test *p* < 0.0001, CT interquartile range (IQR) = 3, CXR IQR = 1). Therefore, the FIGO score derived using CT was significantly higher compared to CXR (Mann–Whitney test *p* = 0.001) (Fig. 1 and Supplementary Fig. S1). Using CT, the FIGO score would have been different in 195 (33.1%) cases, increasing in 188 patients (96.4%) by a median of 1 point (IQR 1–3, maximum 4 points) and decreasing in 7 patients (3.6%) by a median of 1 point (IQR 1–2, maximum 2 points). This would have affected the categorisation of patients into low- or high-risk groups (McNemar’s test, *p* < 0.001) (Table 1), with CT reclassifying 43 (7.3%) patients from the low- to high-risk group.

**Table 1:** Number of low- and high-risk patients predicted using CXR versus CT chest derivation of the FIGO score (McNemar’s test *p* < 0.001, *n* = 589).

	CT	
	LR	HR
**CXR**		
**LR**	475	43
**HR**	0	71

*CXR* chest X-ray, *CT* computerised tomography (chest), *LR* low-risk of single-agent chemotherapy resistance, *HR* high-risk of single-agent chemotherapy resistance.

### Impact upon treatment decisions and patient outcome

**Fig. 2:**
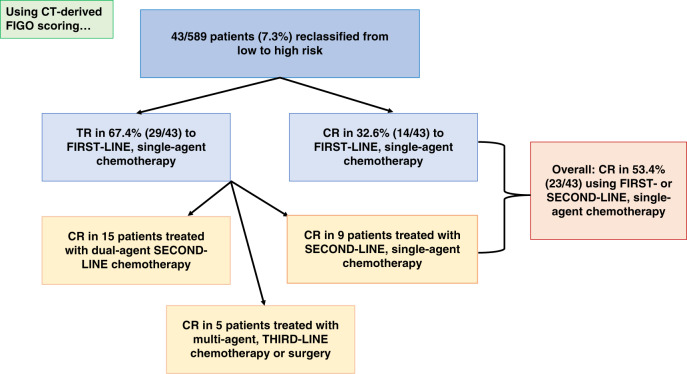
Flow diagram of the treatment outcomes for the 43 patients who changed from low- to high-risk using CT-based pulmonary imaging. All patients ultimately had a CR and survived. *CT* computerised tomography (chest), *TR* resistance to single-agent chemotherapy, *CR* complete response to single-agent chemotherapy.

All treatment decisions were based on CXR alone. However, if CT had been used, of the 43 patients who would have been re-classified from the low- to high-risk group, 14 (32.6%) had CR, and 29 (67.4%) demonstrated TR to single-agent chemotherapy (Fig. 2). All received methotrexate based on their original score.

Of the 29 patients who had TR to single-agent chemotherapy, 9 were cured with second-line, single-agent chemotherapy (dactinomycin *n* = 8, carboplatin n = 1). Therefore, despite being changed from the low- to high-risk group, 23 (53.5%) of the 43 patients achieved a cure with first- or second-line, single-agent chemotherapy.

The remaining 20 patients with TR to single-agent chemotherapy required multi-agent second- (*n* = 15) or third-line (*n* = 5) chemotherapy or surgery (total abdominal hysterectomy) to achieve a cure (Fig. 2).

### Observed incidence of single-agent chemotherapy resistance

The incidence of TR to single-agent chemotherapy was significantly higher among patients whose FIGO score would have changed using CT versus those whose score remained unchanged (Fisher’s exact test *p* = 0.044) (Table 2). The incidence of TR to single-agent chemotherapy was also statistically higher in patients who would have changed from low- to high-risk groups, versus those whose risk did not change (Fisher’s exact test *p* < 0.0001) (Table 3).

**Table 2:** Using CT chest, the breakdown of single-agent chemotherapy response of patients whose FIGO score changed (*n* = 195) versus those whose score remained unchanged (*n* = 394) (Fisher’s exact test *p* = 0.0435, *n* = 589).

	Response to single-agent treatment (% of total)	
	TR	CR
**Score unchanged with CT**	126 (32.0)	268 (68.0)
**Score changed with CT**	79 (40.5)	116 (59.5)

*CXR* chest X-ray, *CT* computerised tomography (chest), *TR* resistance to single-agent chemotherapy, *CR* complete response to single-agent chemotherapy.

**Table 3:** Using CT chest, breakdown of single-agent chemotherapy response of patients whose FIGO category changed from low- to high-risk (*n* = 43) versus those whose risk category remained unchanged (*n* = 546) (Fisher’s exact test *p* < 0.0001, *n* = 589).

	Response to single-agent treatment (% of total)	
	TR	CR
**Risk category unchanged with CT**	176 (32.2)	370 (67.8)
**Risk category changed with CT**	29 (67.4)	14 (32.6)

*CXR* chest X-ray, *CT* computerised tomography (chest), *TR* resistance to single-agent chemotherapy, *CR* complete response to single-agent chemotherapy.

### Effect upon time to remission (TNhCG)

Patients with pulmonary metastases identified on CXR had a significantly longer TNhCG: median TNhCG with no metastases on CXR = 174 days versus 201 days with metastases (log-rank Mantel–Cox test *p* = 0.014). However, metastases on CT were not associated with a longer TNhCG: median TNhCG with no metastases on CT = 173 days versus 182 days with metastases (log-rank Mantel–Cox test *p* = 0.088). TNhCG did not differ between patients who would have changed risk category compared to those whose risk remained unchanged: median TNhCG 181 versus 175 days respectively (log-rank Mantel–Cox test *p* = 0.875).

Referring to the larger patient dataset of 1041 patients diagnosed with GTN who required treatment (chemotherapy or surgery other than uterine evacuations), simply performing a CT scan did not affect TNhCG (median TNhCG = 177 days in 640 patients who had a CT chest versus 169 days in 360 patients who did not undergo a CT chest) (log-rank Mantel–Cox test *p* = 0.063).

### Pulmonary metastases detectable only on CT

In 145 (24.6%) patients, pulmonary metastases were detectable only on CT, which was associated with a statistically higher FIGO score compared to patients with a clear CXR (median of 5 versus 4, Mann–Whitney test *p* < 0.0001) or clear CT (median of 5 versus 3, Mann–Whitney test *p* < 0.0001). The FIGO score increased in all 145 patients by a median of 1 point, which would have led to 36 (24.8%) patients being re-classified from the low- to high-risk group. The incidence of TR to single-agent chemotherapy would have been significantly higher among patients who changed from low- to high-risk groups, compared to those whose risk remained unchanged (Fisher’s exact test *p* = 0.0007).

Of the 36 patients who would have changed from low- to high-risk groups, 13 (36.1%) experienced CR to single-agent chemotherapy. The remaining 23 (63.9%) patients had TR, of whom 5 were subsequently cured with second-line, single-agent chemotherapy (dactinomycin). Overall, 18 (50%) patients were cured with first- or second-line, single-agent chemotherapy. The remainder required multi-agent chemotherapy or surgery as second- (*n* = 14) or third-line (*n* = 4) management.

The incidence of TR to single-agent chemotherapy did not differ between patients with metastases detectable only on CT compared to those with a clear CT (Fisher’s exact test *p* = 0.119).

Patients with pulmonary metastases detectable only on CT did not have a longer TNhCG compared to those with a clear CT chest: median TNhCG 177 days versus 173 days, respectively (log-rank Mantel–Cox test *p* = 0.440).

### Prediction of primary chemotherapy resistance

**Fig. 3:**
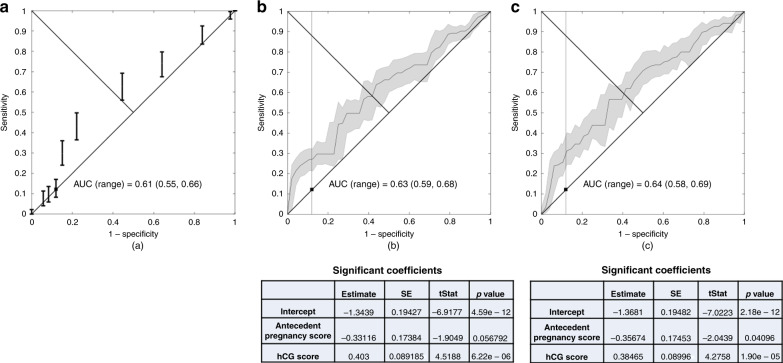
Logistic regression analysis. **a** Using only FIGO score (at a score of 7, sensitivity = 0.12, specificity = 0.88); **b** using the categorised scores from the eight clinical risk factors that constitute the FIGO score, including data derived from CXR chest staging. Matching the specificity achieved by the FIGO score of 7 (0.88), sensitivity is raised to 0.27; ; **c** using the categorised scores from the eight clinical risk factors that constitute the FIGO score, including data derived from CT chest staging. Matching the specificity achieved by the FIGO score of 7 (0.88), sensitivity is raised to 0.31. *CXR* chest X-ray, *CT* computerised tomography (chest), *AUC* area under the curve, *SE* standard error, *tStat* *t* statistic.

The influence of CXR- versus CT-derived FIGO score on the prediction of TR to primary chemotherapy was compared using binomial logistic regression analyses. As a baseline, the capacity of the FIGO score (derived using standard CXR-based chest imaging) to predict TR to primary chemotherapy was poor, with an area under the curve (AUC) of 0.61. For a FIGO score of 7 (the cut-off score for categorising patients as low- versus high-risk), the model had a sensitivity of 0.12 and specificity of 0.88 (Fig. 3a).

Further analyses were conducted using the categorised data from the eight clinical risk factors that constitute the FIGO score. Comparing the predictive models derived from them using either CXR- (Fig. 3b) or CT- (Fig. 3c) based chest imaging revealed a slight, but non-significant improvement to the AUC (AUC = 0.63 versus 0.64, respectively). Despite the small change to the overall AUC, the shape of the ROC curves for both datasets were superior to the baseline curve, particularly in the low false-positive/sensitivity range. This is reflected in the superior sensitivity values when matching the specificity achieved by a FIGO score of 7, with a sensitivity of 0.27 using CXR data (Fig. 3b) versus 0.31 using CT data (Fig. 3c). In summary, combining the categorised scores from the eight clinical risk factors in a logistic regression model, as opposed to using only the FIGO score allows the identification of an additional 15 (CXR-based chest assessment) or 19 patients (CT based chest assessment) who would have TR to primary chemotherapy.

Investigating the eight FIGO risk factors more closely, only two were predictive of primary chemotherapy resistance. Within both CXR and CT chest derived logistic models, the most significant factor was hCG score (*p* < 0.001), with antecedent pregnancy next (*p* < 0.05 for CT and *p* < 0.06 for CXR models) (Fig. 3b, c).

### Incidence of relapse and death

Median follow-up from the date of evacuation was 51.7 months (IQR = 18.0–70.2 months). A total of 18 patients relapsed. The incidence of relapse was unaffected by the presence of pulmonary metastases detected on CXR (Fisher’s exact test *p* = 0.189, *n* = 589) or CT chest (Fisher’s exact test *p* = 0.224, *n* = 589) (Supplementary Table S3). Three patients died from GTN. Of these, one patient had pulmonary metastases detected on CXR, while two patients had metastases on CT chest.

## Discussion

The use of CT chest over CXR in the assessment of GTN is historically controversial. CT would detect more chest metastases compared to CXR; increasing the FIGO score and changing the risk category in a proportion of patients. CT would have improved the prediction of patients who were resistant to single-agent chemotherapy, but crucially would not have improved the outcome for these patients. Overall, the use of CT would not improve the prediction of primary chemotherapy resistance in the whole treated cohort. Performing a CT chest, or the presence of pulmonary metastases on CT were not associated with a longer TNhCG, unlike metastases detected on CXR. Equally, the incidence of relapse was unaffected by the presence of metastases on CXR or CT.

Using CT chest, 7.3% patients would have changed from low- to high-risk, with a statistically higher rate of TR to single-agent chemotherapy, compared to patients whose risk did not change, in agreement with the findings of Price et al.^9^ but dissimilar to an earlier study by Darby et al.^7^ This may be explained by the smaller patient numbers in the latter study. In our study, a significant proportion (53.5%) of the patients who changed risk category would have been over-treated and unnecessarily subjected to the more potential extensive physical, psychological, and longer-term side effects associated with high-risk chemotherapy regimens such as EMA-CO.^17,19^ These figures are in agreement with previous literature comparing CT- versus CXR-derived FIGO scores, whereby 8.3–10.4% patients changed risk category and 50–55% of these responded to single-agent chemotherapy.^7,9^ Given the young patient population affected by GTN, and the frequent desire for further pregnancies, avoiding overtreatment is essential, as is the need to minimise the radiation dose. Despite technological advancements, CT chest delivers 7 millisieverts (mSv) of radiation, equivalent to ~1065 days of natural background radiation exposure. The radiation dose is 350 times higher than a standard postero-anterior CXR, which delivers 0.02 mSv radiation, equivalent to 3 days of background radiation.^20^ Even low-dose CT used for lung cancer screening delivers ~1.4 mSv radiation; 70 times higher than CXR.^21^ Pregnant breast tissue is highly susceptible to radiation,^22^ which applies to GTN patients (all of whom have a raised hCG level), with an increased long-term risk of breast cancer.^23^ Moreover, the financial implications of performing routine CT pulmonary assessment must also be considered, particularly in lower-income countries, where the prevalence of GTN is higher compared to the United Kingdom.^24^ Access to and the increased cost of CT compared to CXR could prevent consistent and comparable investigation of GTN patients across the world.

Metastases detected on CXR were found to be associated with an extended TNhCG, supporting the literature in low-risk patients, suggesting that pulmonary metastases present at the start of treatment are associated with higher rates of TR^7,13,15,18^ and disease recurrence.^16,18^ Metastases detected on CT were not associated with a longer TNhCG.

In the secondary, separate analysis of patients with pulmonary metastases detectable only on CT chest, 24.8% would have changed from the low- to high-risk category, and had a statistically higher rate of TR to single-agent chemotherapy compared to those who did not change risk group. However, similar to patients with pulmonary metastases detectable on both CXR and CT, 50% had a CR to single-agent, first- or second-line chemotherapy and would have been over-treated using CT-derived assessment. Crucially TR to single-agent chemotherapy or TNhCG would not have differed between patients with metastases detectable only on CT compared to those with a clear CT, in agreement with previous literature,^10,14^ but in disagreement with one of the earliest studies by Mutch et al.^11^ The discrepancy with our findings is likely to be explained by the demonstrably larger patient population included within our study, and the improving resolution of modern CT imaging, which can more accurately classify small benign versus malignant chest lesions. Several previous studies included patients with metastases detectable only on CT within their main analyses of metastatic versus non-metastatic disease, hence it is impossible to deduce accurate conclusions regarding their true prognostic significance.^7,9,13,15,16^

Additional secondary analyses revealed that the incidence of relapse was unaffected by pulmonary metastases detected on CXR and CT. A similar analysis could not be performed upon the incidence of death due to the small numbers within the dataset (*n* = 3). Unfortunately, previously published literature comparing CXR and CT did not study these outcome measures.^7,9,13^ Frijstein et al.^18^ demonstrated higher rates of disease recurrence among low-risk patients with pulmonary metastases, compared to those without pulmonary metastases. However, as our study included both low- and high-risk patients, the two studies cannot be compared. Similarly, other literature showing higher rates of relapse among patients with lung metastases^16^ only analysed those with single-site (lung) metastases and excluded patients with metastases at other sites. Our study included patients with both pulmonary and extra-pulmonary metastases at initial assessment.

With regard to multivariate analysis of all FIGO 2000 scoring variables using either CXR or CT, hCG level and antecedent pregnancy were the most important factors for predicting primary treatment resistance, confirming that the use of CT chest did not confer a major prognostic benefit. This conflicts with previously published literature, indicating that metastases on CT chest were the most significant predictor for TR on both uni- and multivariate analysis.^13^ However, that study analysed only six of the eight risk factors within the FIGO system and involved a much smaller patient cohort (*n* = 139).

This study incorporates a large dataset from one of the leading Trophoblastic Centres within the United Kingdom. Limitations of this study include the retrospective analysis, changes, and advances in CT imaging (protocols, slice thickness, resolution) during the time period under study, potentially allowing the detection of smaller pulmonary metastases and improved differentiation of metastatic compared to non-metastatic lesions on more contemporaneous images. One approach would have been to analyse only images taken over the past decade; however, this would have dramatically reduced the sample size and power of the study. Previous studies^25^ have raised concerns that small pulmonary lesions detectable on modern-day CT imaging may, in fact, represent trophoblastic emboli seen even in healthy pregnancies, rather than metastatic GTN, again leading to overtreatment and the unnecessary exposure of patients to more toxic chemotherapy regimens. However, it is impossible to differentiate between such lesions as both resolve over time with or without chemotherapy, while it would be harmful to expose patients to repeated CT chest imaging to monitor the change of such lesions.

Weighing the pros- and cons of CT- versus CXR-derived pulmonary imaging in the assessment of GTN, this study does not support the use of chest CT. CXR should remain the recommended modality of choice for imaging pulmonary metastases as part of FIGO score. The higher radiation dose; increased cost; lack of influence on outcome or prognostic measures render the routine introduction of CT chest in the assessment of GTN patients unnecessary. Furthermore, this study raises questions concerning whether CT chest should be performed even in the instance of an indeterminate CXR, given the lack of evidence to suggest that pulmonary metastases only present on CT, or indeed performing a CT at all, influence any of the key outcome measures studied herein.

## Supplementary information

Supplementary Material

## Data Availability

The datasets used and/or analysed during the current study are available from the corresponding author on reasonable request.
