# Supplementary material for: Computed tomography chest imaging offers no advantage over chest X-ray in the initial assessment of gestational trophoblastic neoplasia
Source: Br J Cancer. 2020 Dec 16;124(6):1066–71. doi: 10.1038/s41416-020-01206-8 (PMC7961138; doi:10.1038/s41416-020-01206-8)
Supplement: Supplementary file 1 — Supplementary Material [file 41416_2020_1206_MOESM1_ESM.docx]

**Supplementary Material**

**Table S_1._ Low- and high-risk treatment regimens at The Sheffield Trophoblastic Centre**

**Abbreviations:** ; hCG, human chorionic gonadotrophin, IU/L, international units per litre.

| **Treatment** | **Regimen description** |
| --- | --- |
| **Low-risk 1st line** | **Methotrexate (MTX): 50mg given intramuscularly every 48 hours for 4 doses, with oral folinic acid given 30 hours after MTX, i.e. MTX on days 1,3,5,7 and folinic acid on days 2,4,6,8. Course repeated every 2 weeks.** |
| **Low-risk 2nd line (hCG <300 IU/L or hCG >300 but <3000 IU/L)** | **Actinomycin D: intravenous bolus at a dose of 1.25mg/m^2^ repeated every two weeks.** |
| **Low-risk 2nd line (hCG >3000 and <30,000 IU/L)** | **Carboplatin (AUC4) intravenous repeated every 2 weeks.** |
| **Low-risk 2nd line (hCG >30,000 IU/L)** | **EA (Etoposide/Actinomycin D): E:100 mg/m^2^ given intravenous, days 1–3, A:0.5 mg intravenous, days 1–3, involving a two night hospital stay, every 10-days.** |
| **Low-risk 3rd line (after MTX and Carboplatin)** | **EA (Etoposide/Actinomycin D): E:100 mg/m^2^ given intravenous, days 1–3, A:0.5 mg intravenous, days 1–3, involving a two night hospital stay, every 10-days.** |
| **High-risk** | **EMA/CO: EMA: Day 1 [Actinomycin: 0.5mg intravenous bolus followed by Etoposide 100mg/m2 in 500mL normal saline as a 1 hour infusion, followed by MTX 300mg/m^2^ intravenous over 12 hours in 1L normal saline], Day 2 [Actinomycin D 0.5mg given as an intravenous bolus followed by Etoposide 100mg/m^2^ in 500mL normal saline as a 1 hour infusion, folinic acid 15mg 6 hourly, given intravenous or orally, 24 hours after the start of MTX. Eight doses are administered]. CO: Day 8 [Vincristine 0.8mg/m^2^ given intravenous in 50mL normal saline over 10 minutes, followed by Cyclophosphamide 600mg/m^2^ given intravenous in 250mL normal saline over 30 minutes].** |

**Table S_2_. Descriptive statistics of the dataset**

**Abbreviations:** IQR, Interquartile range, data presented as 25^th^ and 75^th^ percentile; mm, millimetres; IU/L, international units per litre; hCG, human chorionic gonadotrophin; FIGO, International Federation of Gynecology and Obstetrics; CXR, chest X-ray; CT, computerised tomography (chest).

|  |  |  |  |  |  | **CXR derived data** | | | **CT derived data** | | |
| --- | --- | --- | --- | --- | --- | --- | --- | --- | --- | --- | --- |
|  | **Maternal Age (years)** | **Interval (months)** | **hCG prompting treatment (IU/L)** | **FIGO Stage** | **Time to normal hCG level (days)** | **Largest tumour size (mm)** | **Total number of metastases** | **FIGO**  **score** | **Largest tumour size CT (mm)** | **Total number of metastases** | **FIGO**  **score** |
| **Median** | 28.97 | 2.61 | 13,948 | 1 | 175 | 40 | 0 | 4 | 40 | 0 | 4 |
| **IQR** | 23.88 to 35.04 | 1.61 to 4.21 | 2791 to 59,481 | 1 to 3 | 143 to 227 | 12 to 60 | 0 to 1 | 2 to 5 | 15 to 60 | 0 to 3 | 2 to 6 |
| **Minimum** | 14.68 | 0.18 | 7 | 1 | 59 | 0 | 0 | 0 | 0 | 0 | 0 |
| **Maximum** | 56.62 | 135.80 | 1,454,810 | 4 | 6475 | 150 | 19 | 21 | 150 | 70 | 21 |

**Table S_3._ Incidence of relapse (n=18) in patients with pulmonary metastases detected on CXR (Fisher’s exact test p=0.189, n=589) or CT chest (Fisher’s exact test p=0.224, n=589).**

**Abbreviations:** CXR, chest X-ray; CT, computerised tomography (chest).

|  | **Relapse** | **No relapse** |  | **Relapse** | **No relapse** |
| --- | --- | --- | --- | --- | --- |
| **Pulmonary metastases detected on CXR** | 5 | 90 | **Pulmonary metastases detected on CT** | 10 | 228 |
| **No pulmonary metastases detected on CXR** | 13 | 481 | **No pulmonary metastases detected on CT** | 8 | 343 |

**Figure S_1_. Histogram comparing of the FIGO scores calculated using CXR versus CT chest. The green threshold line delineates a FIGO score of 7; the cut-off for categorising patients as low- versus high-risk of single-agent chemotherapy resistance.**

Abbreviations: FIGO, International Federation of Gynecology and Obstetrics; CXR, chest X-ray; CT, computerised tomography (chest).
